# Thyroid function disorders and secondary cancer following haematopoietic stem cell transplantation in pediatrics: State of the art and practical recommendations for a risk-based follow-up

**DOI:** 10.3389/fendo.2022.1064146

**Published:** 2022-12-21

**Authors:** Alessandro Cattoni, Silvia Molinari, Benedetta Riva, Santo Di Marco, Marta Adavastro, Martha Caterina Faraguna, Vittoria Garella, Francesco Medici, Maria Laura Nicolosi, Claudia Pellegrinelli, Martina Lattuada, Donatella Fraschini, Fabio Pagni, Andrea Biondi, Adriana Balduzzi

**Affiliations:** ^1^ Department of Pediatrics, Università degli Studi di Milano-Bicocca, Fondazione Monza e Brianza per il Bambino e la sua Mamma (MBBM), Ospedale San Gerardo, Monza, Italy; ^2^ Department of Pathology, Università degli Studi di Milano-Bicocca, Ospedale San Gerardo, Monza, Italy

**Keywords:** thyroid disorder, haematopoietic stem cell transplantation (HSCT), busulfan, total body irradiation (TBI), thyroid malignancy, childhood cancer survivors (CCS), long-term follow-up

## Abstract

Thyroid disorders (TD) represent a remarkable share of all the late morbidities experienced following pediatric haematopoietic stem cell transplantation (HSCT), with long-term reported occurrence often exceeding 70%. In addition, the data collected on wide cohorts of survivors assessed longitudinally outlined a progressive increase in the cumulative incidence of TD as far as 30 years following transplantation. Accordingly, a life-long monitoring of thyroid health is warranted among patients exposed to HSCT in childhood, in order to early detect TD and undertake a prompt dedicated treatment. Although several national and international *consortia* have provided recommendations for the early detection of thyroid disorders among childhood cancer survivors exposed to radiotherapy and alkylating agents, no guidelines specifically and thoroughly focused on HSCT-related TD have been published to date. As stem cell transplantation has become the standard-of-care in a growing body of non-oncological conditions, this urge has become pivotal. To highlight the challenging issues specifically involving this cohort of patients and to provide clinicians with the proposal of a practical follow-up protocol, we reviewed published literature in the light of the shared experience of a multidisciplinary team of pediatric oncologists, transplantologists, pathologists and endocrinologists involved in the long-term care of HSCT survivors. As a final result, we hereby present the proposals of a practical and customized risk-based approach to tailor thyroid health follow-up based on HSCT-related detrimental factors.

## 1 Introduction

In the last decades, haematopoietic stem cell transplantation (HSCT) has become the standard-of-care in the treatment of a growing body of clinical conditions, encompassing malignant and non-malignant hematological disorders, inborn errors of metabolism and congenital immunodeficiencies (1). More than a million and a half HSCT have been reported over the last 6 decades (2) and even though early- and late-onset complications still expose patients to a remarkable risk of morbidity and mortality, survival rates in children, adolescents, and young adults have significantly increased as a consequence of the improvement in histocompatibility matching, donor selection procedures, supportive therapy and tailored multidisciplinary approach (3–5). Accordingly, the overall burden of late-onset morbidities has increasingly become a pivotal part of the long-term care of HSCT-survivors.

In pediatric HSCT, endocrinopathies represent a big share of late morbidities, affecting over 60% of long-term survivors undergoing HSCT before the age of 10 years (6). Thyroid disorders (TD), with cumulative incidences as high as 73%, are among the most frequently reported late effects following HSCT.

Transplantation-related TD include central hypothyroidism, non-autoimmune impaired thyroid function (subclinical or overt primary hypothyroidism), autoimmune thyroid disease (Hashimoto thyroiditis, Graves’ disease), volumetric and parenchymal changes, and benign and malignant nodules (7, 8).

In literature both treatment- and host-related risk factors for TD have been assessed. Among the latter, younger age upon transplantation and underlying genetic syndromes (i.e. Fanconi anemia) play an independent detrimental role on thyroid health following transplantation (9, 10). Conversely, treatment-related determinants include: antineoplastic therapy provided before HSCT (i.e. mainly locoregional radiotherapy involving the neck, the mediastinum or the head and, to a lower extent, systemic chemotherapy), prolonged exposure to immunosuppressants, graft-versus-host disease, prolonged administration of tyrosine kinase inhibitors (TKI) in *Philadelphia chromosome* positive leukemias and the conditioning provided before HSCT (11).

The term “conditioning” indicates the preparative treatment administered prior to HSCT with the purpose of reducing the tumor burden, in case of a malignant disorder, and allowing donor stem cell engraftment. Based on clinical indication and host-related features, the conditioning regimen consists of either chemotherapy alone or a combination of chemo- and radiotherapy (total body irradiation, TBI).

Given the extreme susceptibility of thyroid tissue to radiation, the deleterious effects of TBI as well as cranial or mediastinal locoregional radiation on the developing gland has been extensively assessed. On the other hand, though a growing body of evidence has demonstrated the detrimental effects also of TBI-free conditionings on thyroid health, these data are mostly drawn from retrospective analyses held on small cohorts of HSCT survivors and the specific role of several alkylating agents has not been fully assessed yet (9, 12, 13).

Finally, the risk of developing a secondary thyroid malignancy is remarkably increased following HSCT compared to healthy controls, hence representing a significant determinant of the burden of morbidity among transplanted survivors.

The purpose of this analysis is to perform an extensive revision of published literature about incidence, specificities and risk factors of thyroid late effects following HSCT, with the ultimate purpose of providing clinicians with practical and risk-based recommendations for the management of TD in the setting of long-term follow-up.

## 2 Thyroid dysfunction and autoimmune disorders

### 2.1 The epidemiology of thyroid function disorders following HSCT

Primary hypothyroidism, either non-autoimmune or in the setting of Hashimoto thyroiditis, represents the most frequent thyroid function disorder following HSCT, with reported prevalence ranging from 10 to 50% and with most of this variability being due to differences in the composition, baseline HSCT indication, follow-up duration and conditioning regimen administered (12, 14–17). As a result of a 20-year systematic endocrine follow-up held on a cohort of 244 survivors transplanted during childhood or adolescence in our institution, we have previously reported hypothyroidism with a prevalence of 17.2% (non-autoimmune 14.3%; autoimmune 2.9%) and with 10-year and 15-year cumulative incidences as high as 22.5% and 34.0%, respectively (18). These data are consistent with those reported by other authors in the setting of late effects assessment (19, 20).

The occurrence of autoimmune hyperthyroidism following HSCT has been mostly described in the setting of single reports or case series (21–25). Few wider cohorts have reported the prevalence of Graves’ disease following pediatric HSCT, ranging from in 0.8 to 2.9% (18, 26). Whether patients exposed to high-dose radiotherapy involving the neck or the mediastinum prior to conditioning and HSCT were included or not explains most of the reported incidence variability in these series.

Several factors related to HSCT may interact and result in thyroid dysfunction, such as the exposure to radiotherapy, alkylating agents, novel targeted therapy such as TKI, and altered immune reconstitution following HSCT.

**Table 1** lists all the thyroid disorder experienced following HSCT and the relative risk factors and prevalence.

**Table 1:** Thyroid disorders following stem cell transplantation: risk factors, occurrence rates and specificities among HSCT survivors conditioned with TBI-based or TBI-free regimens.

Thyroid disorder following pediatric HSCT	Risk factors and related pathogenesis	Diagnosis	Prevalence/incidence after TBI-based conditioning	Prevalence/incidence after chemo-only conditioning	Peculiarities of clinical presentation among transplanted patients
**Non-autoimmune hypothyroidism**	***Total body or loco-regional irradiation*** *Highest risk*: thyroid dosimetry > 25 Gy *Pathogenesis*: - direct thyroid damage: cytotoxicity and apoptosis - thyroid hypoplasia ***Chemotherapy-based conditioning*** *Highest risk*: alkylating agents (Busulfan, Cyclophosphamide), bleomycin *Pathogenesis*: direct thyroid damage ***Thyrosine-Kinase inhibitors*** *Pathogenesis:* - inhibition of thyroid peroxidase activity - interference with the pituitary feedback - inhibition of T4 deiodination - impaired iodide update - accelerated T4 and T3 clearance - VEGF inhibition ***^131^I-MIBG*** *Pathogenesis:* direct thyroid damage. ***Immunotherapy (interferon-α, interleukin-2)*** *Pathogenesis:* direct thyroid damage.	**Screening investigations:** ***TSH, freeT4, freeT3 Neck palpation*** Periodically assessed based on patient’s specific risk factors (See **Table 2**). **Additional investigations in case of abnormal findings at screening phase (raised TSH, palpable goiter):** ***A. Anti-thyroid peroxidase and anti-thyroglobulin antibodies*** Not performed routinely, but: -Assessed at first detection of hypothyroidism in order to rule out autoimmune thyroiditis - in case of sonographic signs consistent with thyroiditis ***B. Thyroid ultrasound, in order to:*** - rule out signs consistent with thyroiditis - show congenital or acquired anatomic variants (hypoplasia after TBI; emi-hypoplasia in case of monolateral radiotherapy) - detect coexistent nodules	***TBI -*** Prevalence: 17-37% 10-year cumulative incidence: 26 to 50%	***Busulfan -*** Prevalence: 7-20% 5-year cumulative incidence: 12-15% **Other chemo-only regimens** Prevalence: 2-8% 5-year cumulative incidence: 3-5%	***Symptoms ascribable to hypothyroidism may be overestimated*** due to the co-occurrence of multiple endocrine deficiencies (hypogonadism, growth hormone deficiency), psychological issues or diseases involving different systems (restrictive respiratory syndrome, cardiac failure)
**Hashimoto thyroiditis**	***Total body or loco-regional irradiation*** *Highest risk*: thyroid dosimetry > 25 Gy *Pathogenesis*: damage resulting in the presentation of otherwise unexposed thyroid antigens to the immune system ***Chemotherapy-based conditioning*** *Highest risk*: alkylating agents (Busulfan, Cyclophosphamide), bleomycin *Pathogenesis*: damage resulting in the presentation of otherwise unexposed thyroid antigens to the immune system ***Graft versus host disease (GvHD)*** *Debated*: conflicting outcomes about the role of GvHD *per se*, as its role is mostly deemed as mediated by treatment of GvHD with immunosuppressants ***Prolonged administration of immunosuppressants*** ***^131^I-MIBG*** *Pathogenesis:* damage resulting in the presentation of otherwise unexposed thyroid antigens to the immune system ***Immunotherapy (interferon-α, interleukin-2)*** *Pathogenesis:* damage resulting in the presentation of otherwise unexposed thyroid antigens to the immune system	**Screening investigations: *TSH, freeT4, freeT3 Neck palpation*** Periodically assessed based on patient’s specific risk factors (See **Table 2**). **Additional investigations in case of abnormal findings at screening phase** **(raised TSH, palpable goiter):** ***A. Anti-thyroid peroxidase and anti-thyroglobulin antibodies*** Not performed routinely, but: -Assessed at first detection of hypothyroidism - in case of sonographic signs consistent with thyroiditis ***B. Thyroid ultrasound, in order to:*** - confirm signs consistent with thyroiditis - detect coexistent nodules	***TBI -*** Prevalence: 5-8%	***Busulfan -*** Prevalence: 2-4%	***Adoptive transfer of autoantibodies from SCT donor to recipient may occur*** Patients may show positive antibodies but normal ultrasound, hence challenging the diagnosis of thyroiditis and supporting the hypothesis of antithyroid antibodies secretion by non- pathogenic lymphocytes after stem cell engraftment
**Graves’ disease**	***Total body or locoregional radiotherapy involving the gland.*** *Highest risk*: - locoregional RT delivering > 35 Gy to the thyroid, i.e. Hodgkin Lymphomas or mediastinum/neck solid tumors (dose-dependent effect) - craniospinal irradiation *frontline* - cranial irradiation *frontline* plus TBI *Pathogenesis* Thyroid damage resulting in the presentation of otherwise unexposed thyroid antigens to the immune system, with subsequent synthesis of antiTSH-receptor antibodies ***Graft versus host disease (GvHD)*** Debated: conflicting outcomes about the role of GvHD *per se*, as its role is mostly deemed as mediated by the need for prolonged administration of immunosuppressants ***Prolonged immunosuppression*** ***GvHD prophylaxis with anti-thymocyte globulin (ATG) or alemtuzumab*** *Pathogenesis: T-cell depletion by cytolysis, thus leading to massive release of chemokines and cytokines and subsequent inflammatory response that may involve the thyroids. This results in exposure of thyroid antigens.* ***Immunotherapy (interferon-α, interleukin-2)*** *Pathogenesis:* damage resulting in the presentation of otherwise unexposed thyroid antigens to the immune system.	**Screening investigations: *TSH, freeT4, freeT3 Neck palpation*** Periodically assessed based on patient’s specific risk factors (See **Table 2**). **Additional investigations in case of abnormal findings at screening phase (suppressed TSH, raised FT4/FT3, goiter):** ***A. AntiTSH-receptor, anti-thyroid peroxidase and anti-thyroglobulin antibodies*** ***B. Thyroid ultrasound***	Prevalence: 0.5-2%	Prevalence: 0-7% (includes patients treated with ATG and Alemtuzumab)	***Adoptive transfer of autoantibodies from SCT donor to recipient may occur*** Pathogenic phenomenon. Several cases of SCT recipients developing Graves’ disease following the onset of the disease in familiar donors. ***Theoretically, the frailty of HSCT survivors may magnify the risk of developing anti-thyroid medications side effects.*** In detail: agranulocytosis and abnormal liver function tests may occur more frequently, though no data have been published about this topic.
**Central hypothyroidism (TSH deficiency)**	***Hypothalamic-pituitary (HP) irradiation*** *Highest risk*: HP dosimetry > 30 Gy: - TBI in a patient treated with prophylactic cranial or craniospinal therapy *frontline* (ALL) *Pathogenesis*: hypothalamus is radio-sensitive. ***Bexarotene (Cutaneous T-Cell Lymphoma)*** *Pathogenesis:* pituitary TSH secretion is suppressed.	**Screening investigations: *TSH, freeT4, freeT3*** Periodically assessed based on patient’s specific risk factors (See **Table 2**). ***Whole pituitary function assessment in patients with confirmed central hypothyroidism*** *Indication and frequency based on radiation dosimetry*	*Prevalence:* *0% after TBI only;* *1-9% among patients treated before HSCT with cranial or craniospinal prophylaxis for CSN ALL involvement.*	*NA*	***Infrequent finding among HSCT survivors.*** Dedicated screening is recommended for ALL patients treated with cranial or craniospinal prophylaxis *frontline* due to the risk of SNC disease involvement, subsequently conditioned with TBI before HSCT (cumulative HP dosimetry ≥ 30 Gy).
**Thyroid nodules (benign and malignant)**	***Total body or loco-regional irradiation*** *Highest risk*: - thyroid dosimetry 20-29 Gy; decreasing risk for doses > 30 Gy. - age at irradiation <10 years *Pathogenesis*: radiation-induced DNA damage and subsequent oncogenesis-promoting gene mutation. ***Chemotherapy-based conditioning:*** debated, results not conclusive due to paucity of the samples. *Highest risk*: alkylating agents (Busulfan, Cyclophosphamide) *Pathogenesis*: DNA damage and gene mutation **GvHD:** debated, conflicting outcomes	**Screening investigations: *Neck palpation or thyroid ultrasound*** periodically assessed according to risk factors – see **Table 2** and paragraph 4 for additional information about the most fitting choice **Additional investigations: *fine needle aspiration biopsy (FNAB)*** In case of suspected malignancy (according to radiologic criteria combined to clinical risk factors)	**Nodules (either malignant or benign)** *Prevalence:* 16.4-60% *15-year cumulative* incidence: 66.8% **Thyroid cancer** *Prevalence*: 0-14% *10-year cumulative incidence:* 8%	**Nodules (either malignant or benign)** *Prevalence:* 11% *Prevalence following Busulfan:* 12.2% **Thyroid cancer** *Prevalence:* 0-2%	***Papillary hystotype*** *is the most frequently found among irradiated patients.* ***Secondary thyroid cancers are typically infiltrative tumors*** *, with a higher degree of dedifferentiation and a more aggressive clinical behavior, as a result of frequent rearrangements in oncogenes.* ***No association between functional and structural disorders*** ***The size of nodule may not be a reliable parameter*** to discriminate between a lesion deserving FNAB or not among HSCT survivors.

VEGF, Vascular Endothelial Growth Factor; ^131^I-MIBG, ^131^I-MIBG - iodine-131 meta-iodobenzylguanidine; TBI, total body irradiation; GvHD, graft versus host disease; CNS, central nervous system; ALL, acute lymphoblastic leukaemia; FNAB, fine needle aspiration biopsy; ATG, antithymocyte globulin.

### 2.2 Risk factor for thyroid function disorders following HSCT

#### 2.2.1 Total body irradiation

TBI represents the cornerstone of the conditioning regimen in pediatric patients with acute lymphoblastic leukemia (ALL) and excellent overall and disease-free survival rates have been demonstrated for children with high-risk ALL conditioned with TBI and etoposide before HSCT from an HLA matched donor (27–31).

TBI consists of a homogenous dose of ionizing radiation uniformly distributed over the entire body, in order to eradicate leukemic cells and to enable stem cell engraftment by suppressing immune rejection in the recipient.

In the past, myeloablative radiotherapy was provided as a single-pointed fraction (up to 10 Gy). In spite of the logistical advantage, such an approach resulted in a remarkable burden of multisystemic early- and late-onset adverse events. By applying radiobiology modeling in the field of radioprotection, several studies have demonstrated that delivering the overall dosimetry as reduced dose-fractions was able to minimize the overall toxicity, though providing equal or improved survival rates (32–35). Accordingly, in order to improve the therapeutic *ratio* of radiosensitive healthy tissues *versus* the immunosuppressive and anti-leukemic effects of TBI, fractionated total body irradiation has currently become the standard-of-care, with 12 Gy in 6 fractions delivered over 3 days being the most common schedule (35).

When a patient undergoes TBI, the whole hypothalamic-pituitary axis falls within the radiation field and the disruption of any of the elements of the TRH-TSH-thyroid loop may result in primary or secondary thyroid dysfunction (16).

Primary hypothyroidism is the most frequently reported benign thyroid dysfunction following TBI in childhood. Indeed, the developing gland has been demonstrated to show dramatic sensitivity to the long-term effects of radiation exposure, as proven in studies of children exposed to sublethal radiation doses for nuclear catastrophes (36). Consistently, younger age upon transplantation (< 10 years) is a universally recognized risk factor for the development of subsequent thyroid dysfunction (17, 26, 37–39).

The reported 10-year cumulative incidence of either autoimmune or non-autoimmune primary hypothyroidism in cohorts of patients conditioned with TBI in childhood or adolescence for ALL ranges from 26 to 50%, with most of this variability depending on the decision to either include or exclude those patients previously exposed to cranial prophylactic radiotherapy as a part of front-line therapy prior to conditioning (15, 18, 19, 38).

From a pathogenic perspective, several radiation-dependent mechanisms may interplay in affecting thyroid function. Firstly, a direct damage involving thyrocytes and extracellular tissue occurs according to a dose-dependent model and may result in gland atrophy, with cells and tissues losing volume and function as thyroid dosimetry increases (40). Atrophy tends to occur early after TBI, with a statistically significant volume reduction being detected 1 year after HSCT and with fewer additional changes occurring subsequently (18). Conversely, the cumulative incidence of hypothyroidism shows a regular and persistent increase in time, supporting the hypothesis of an initially preserved thyroid function despite early atrophy and a slower secretive decline following TBI.

Secondly, it has been suggested that radiotherapy itself may promote the onset of autoimmune thyroid disorders. Indeed, following radiation, autoantigens released from a damaged thyroid may be recognized by the immune system and hence prompt cell-mediated cytotoxicity, resulting in inflammation and secretive dysfunction (41, 42). This mechanism is regarded as stochastic and non-threshold-related.

The pathogenesis of autoimmune hyperthyroidism following radiation is superimposable, but the exposure of otherwise undetectable thyroid antigens to the immune system results in the synthesis of TSH-receptor stimulating antibodies (43). This occurrence is rare after TBI (overall occurring in less than 1% of patients), while the prevalence of Graves’ disease is remarkably higher among patients exposed to higher dosimetry involving the gland, i.e. in case of lymphomas or solid tumors of the neck or the mediastinum treated with locoregional radiotherapy. Though still debated, these data suggest a dose-dependent relationship, with hyperthyroidism occurring for thyroid radiation doses above the threshold of 35 Gy (44). Consistently, in a retrospective report held in the setting of the Childhood Cancer Survivor Study (CCSS) on over 12.000 patients treated for solid or liquid tumors, a linear model between thyroid radiation dose exposure and incidence of hyperthyroidism was outlined (41). Finally, the occurrence of hyperthyroidism among transplanted patients irrespectively of the conditioning regimen undertaken has led several authors to hypothesize the adoptive transfer of immune-related factors from donor to recipient, possibly including anti-TSH-receptor antibodies (45).

Central hypothyroidism is regarded as a rare finding following TBI, as the TRH-TSH axis shows a remarkable resistance to radiation-induced damage and TSH deficiency does not generally occur for the low dosimetry delivered by TBI. Hypothalamic-pituitary exposures above 30 Gy, as in the setting of irradiated CNS solid tumors, represents a theoretical threshold above which the incidence of TSH deficiency becomes clinically significant. The unexpectedly high incidence of central hypothyroidism historically reported by some authors following TBI was based on abnormal responses to the TSH-releasing hormone stimulation test, of which reliability is now questioned (46). In addition, most studies have assessed the cumulative incidence of TSH deficiency among transplanted patients, irrespectively of the previous exposure to prophylactic cranial or cranio-spinal irradiation delivered as a front-line treatment, hence not allowing authors to draw ultimate conclusions about the specific detrimental role of low dosimetry delivered by TBI upon hypothalamic-pituitary function (26, 47). Studies that excluded patients exposed to cranial radiotherapy prior to TBI reported cumulative incidence of central hypothyroidism as low as 0% (48, 49).

#### 2.2.2 Chemo-only conditioning regimens

Although classically associated with TBI-based regimens, an increased occurrence of thyroid function disorders has also been described after chemotherapy only-based conditionings, with reported incidence ranging from 0 to 29% (9, 12, 15, 26, 50–53). Though this hypothesis has been historically challenged by small sample-sized studies (12, 13, 54), a growing body of literature has shed light upon the detrimental role on thyroid health played by high doses of alkylating agents administered as a preparative schedule prior to HSCT (9, 18, 26). Accordingly, studies on either myeloablative or reduced-intensity TBI-free conditioning regimens administered in the setting of both malignant and benign conditions have assessed the occurrence of thyroid dysfunction. Despite a trend towards an apparent greater burden of thyroid disorders following myeloablative regimens, also reduced-intensity chemo-only conditionings (i.e. those used in the field of primary immunodeficiencies) have been demonstrated to result in a negative impact on thyroid health (51, 52).

It may be argued that HSCT itself or other HSCT-related factors may be associated to a statistically significant increase in the occurrence of TD. Nevertheless, firstly, studies performed in animal models have shown a histologically-confirmed follicular damage following exposure to high-dose chemotherapy administered before HSCT, including mitochondrial damage, distention and vacuolization of the endoplasmic *reticulum cisternae* (55). In addition, it can be hypothesized that chemotherapy-dependent thyroid damage may result in the exposure of antigens that are not normally presented to the immune system, hence prompting the onset of dysimmune phenomena and ultimately autoimmune thyroid disease. Finally, remarkably divergent cumulative incidence has been drawn among patients conditioned with different regimens, hence supporting the hypothesis of a causative role of specific agents.

In the CCSS, bleomycin and alkylating agents have been identified as potentially causative factors for iatrogenic hypothyroidism (56).

More recently, high-dose busulfan (Bu) seems to expose patients to an overall greater risk of developing either autoimmune or non-autoimmune thyroid function disorders compared to cyclophosphamide (Cy), fludarabine and other alkylating agents (26). Over a long-standing 30-year follow-up, Sanders and colleagues demonstrated that the combination of Bu and Cy resulted in a remarkably greater cumulative incidence of TD than Cy alone, with thyroid function disorders occurring as long as 10 years after HSCT (26). Accordingly, we recently reported that the 5- and 10-year cumulative incidence of thyroid function disorders did not differ statistically between TBI- and Bu-based conditioning regimens, with both of them being remarkably greater than other TBI-free schedules (10-year cumulative incidence: 25.9% (SE 5.0%) for TBI *versus* 22.2% (SE 5.2%) for Bu *versus* 9.8% (SE 4.7%) for other chemo-only regimens). In addition, the 5-year cumulative incidence of non-autoimmune hypothyroidism was statistically higher after Bu-based compared with Treo/Flu/Cy-based conditioning regimens (12.4% versus 3.1%: *p* 0.02) (18).

The potentially lower occurrence of endocrine late effects following treosulfan *versus* busulfan has been extensively investigated in the last decade, but with a specific focus on gonadal function (57). Only recently, two retrospective studies have highlighted that treosulfan seems to show a trend towards a safer toxicity profile on thyroid health, though longer-lasting additional analyses are warranted before this conclusion can be systematically drawn (18, 58).

#### 2.2.3 Graft versus host disease and prolonged administration of immunosuppressants

Graft *versus* host disease (GvHD) is an alloimmune process characterized by activated donor T-cells recognizing host target antigens and cells as non-self and thus mediating cytotoxic tissue damage. This phenomenon shows prevalence rates as high as 40% to 60% among allogeneic HSCT recipients despite the administration of immunosuppressants to prevent GvHD (59). The core of its pathophysiology and the relationship between GvHD and the hazard of developing autoimmune diseases after HSCT has been thoroughly investigated (60). Nevertheless, the association between GvHD and autoimmune thyroid disorders needs to be further clarified. Few published cases reported thyroiditis in the setting of chronic GvHD and these findings have led some authors to speculate that the phenomena of immune reconstitution and dysregulation following GvHD may play a causative role on the pathogenesis of autoimmune thyroid disorders (61, 62). Conversely, most authors have challenged the hypothesis that GvHD may act *per se* as an independent causative factor for the development of autoimmune thyroiditis, whereas conflicting outcomes have been reported about the detrimental role played on thyroid health by immunosuppressants administered to control GvHD (19, 63). In a homogeneous cohort of 97 pediatric ALL patients, Zubarovskaya and colleagues found that neither prolonged immunosuppression nor chronic GvHD were positively associated with TD, while the administration of anti-thymocyte globulin (ATG), used in the early prophylaxis of GvHD, was regarded as a detrimental factor on thyroid function (19). The authors speculated that the gland may be adversely affected by the systemic inflammatory response elicited by complement-dependent T-cell lysis and subsequent massive release of cytokines and chemokines that follows ATG administration. Consistently, higher occurrence rates of Graves’ disease following ATG treatment for GvHD prophylaxis among patients with severe aplastic anemia have been reported (21, 23).

In a cohort of transplanted ALL adult survivors, Savani and colleagues demonstrated a statistically greater occurrence of hypothyroidism among patients with chronic GvHD treated with prolonged immunosuppression. In addition, the authors showed that all the patients with subclinical hypothyroidism who required long-lasting exposure to immunosuppressants for GvHD developed symptomatic hypothyroidism and required hormonal replacement therapy (63).

#### 2.2.4 Coexistent non-HSCT-dependent risk factors

When assessing the hazard of developing thyroid function disorders following HSCT, clinicians need to consider additional risk factors not directly associated to transplantation itself, but recurrent among transplanted patients as a part of the overall diagnostic and therapeutic path.

Firstly, the underlying indication to HSCT should be considered. For example, patients with Fanconi anemia develop hypothyroidism in almost 60% of cases, regardless of HSCT (64). Similarly, the greater prevalence rates of immune dysregulation occurring among patients with primary immunodeficiency results in a risk factor for Graves’s disease and Hashimoto thyroiditis *per se* (65).

Secondly, TKI have extensively become the cornerstone in the treatment of Philadelphia chromosome-positive ALL and chronic myeloid leukemia (CML). TKI-induced hypothyroidism has been identified as a potential side effect, especially in patients with preexisting thyroid dysfunction. Thyroid function disorders have been reported in 25, 55 and 70% of patients diagnosed with CML and treated with imatinib, nilotinib and dasatinib, respectively (11). The pathophysiology of TD following prolonged administration of TKI is debated. Direct inhibition of thyroid peroxidase activity, interference with the pituitary feedback exerted by thyroid hormones, inhibition of T4 deiodination, impaired iodide uptake, accelerated T4 and T3 clearance and capillary regression induced by vascular-endothelial growth factor (VEGF) inhibition are some of the hypothesized mechanisms leading to thyroid dysfunction (66, 67).

In addition, in patients affected with lymphomas or solid tumors involving the neck, the mediastinum or the spine treated with locoregional radiotherapy before HSCT, the thyroid may totally or partially fall within the irradiation field. Similarly, prophylactic cranial irradiation has been a standard treatment in children with acute ALL with high risk for central nervous system relapse. As leukemia is one of the most radiosensitive tumors, the dose administered by cranial radiation is lower than that administered for other neoplasms, with brain dosimetry ranging from 18 to 24 Gy, but with 0.13 to 1.32 Gy delivered to the thyroid gland as scatter irradiation (68). Accordingly, the exposure to locoregional or cranial irradiation in a patient assessed after HSCT results in a remarkably greater risk of developing TD (18, 26, 38).

Furthermore, several immunotherapies adopted as antineoplastic agents (including interferon-α and interleukin-2) have been shown to result in a higher incidence of primary thyroid dysfunction (69, 70). Immunotherapy-induced thyroid dysfunction occurs secondary to thyroiditis, which can be either autoimmune or non-autoimmune. The latter is characterized by a typically asymptomatic thyrotoxic phase, followed by the development of hypothyroidism.

Refractory juvenile cutaneous T-cell lymphoma represents an extremely rare indication to allogeneic HSCT. Bexarotene is a synthetic retinoid X receptor-selective ligand approved for the treatment of this condition, both in adolescence and adulthood. This medication may result in central hypothyroidism as a consequence of the suppression of the pituitary TSH secretion, thus prompting the need for a dedicated screening among survivors.

Finally, pediatric patients with high-risk neuroblastoma (NBL) benefit from high-dose chemotherapy followed by autologous stem cell rescue. Metaiodobenzylguanidine (MIBG) is a guanethidine derivative and analogue of norepinephrine with specific affinity for neural crest tissues. When it is labelled with iodine-131 (^131^I-MIBG), its administration has been demonstrated as effective to treat NBL. Nevertheless, as the result of the physiological uptake of iodine by the thyroid gland, ^131^I-MIBG administration is associated with a remarkable degree of thyroid toxicity, and NBL patients may experience hypo- or hyperthyroidism, regardless of the exposure to autologous HSCT (71, 72). Thyroid protection through potassium iodine, perchlorate, or the combination of potassium iodide, T4, and a thiamazole decreases but does not entirely eradicate the risk of ^131^I- MIBG–induced hypothyroidism (71).

#### 2.2.5 The contribution of different HLA haplotypes

Only very few studies have assessed the genetic predisposition for thyroid disease following hematopoietic stem cell transplantation and the potential role played by different HLA haplotypes. Au et al. reported as significant genetic risk factors HLA B46 and HLA DR9 among 10 patients with autoimmune thyroid disease out of 721 Chinese adult transplanted patients (60).

Moreover, Hawkins et al. reported HLA DR9 as a risk factor for Graves’ disease (73). However, different ethnicities of participants, small sized samples and different results hinder further interpretations in this setting.

### 2.3 Diagnosis and treatment of thyroid function disorders: Peculiarities, lights and shadows among pediatric HSCT survivors

No guidelines specifically focused on diagnosis and treatment of either autoimmune or non-autoimmune thyroid function disorders following transplantation have been published to date. Although diagnostic criteria, indications, dosages and monitoring of hormonal replacement therapy and anti-thyroid medications follow the general principles endorsed for the otherwise healthy population, we believe that the specificities and frailty of transplanted children, adolescents and young adults should lead clinicians to tailor a customized approach.

#### 2.3.1 Hypothyroidism

From a diagnostic perspective, the detection of antithyroglobulin and/or antithyroperoxidase antibodies among HSCT survivors should prompt a complete biochemical and sonographic assessment, including thyroid function tests and thyroid ultrasound. Nevertheless, positive autoantibodies are often deemed as the result of the adoptive transfer of immunological *stigmata* from the donor to the recipient, hence possibly not representing a marker of lymphoid dysregulation and infiltration in the setting of Hashimoto thyroiditis among HSCT survivors (61). Thus, an integrated evaluation of biochemical, autoimmune and sonographic data is pivotal to correctly label either autoimmune or non-autoimmune TD. As in pediatrics the strength of the indication to hormone replacement therapy in case of subclinical hypothyroidism is remarkably affected by an underlying diagnosis of autoimmune chronic thyroiditis, this consideration shows considerable effects on treatment strategies (74).

Furthermore, endocrinologists should be aware that multiple hormonal deficiencies and non-endocrine conditions experienced by HSCT survivors may interplay and result in a pattern of signs and symptoms potentially mimicking hypothyroidism, thus leading to an apparent overestimation of the severity of the clinical burden of mild thyroid dysfunctions. In detail, hypergonadotropic hypogonadism, growth hormone deficiency, cardiac pump defects, radiation- or busulfan-induced respiratory restrictive syndrome and psychological issues may variably compete as causative factors for fatigue, weight gain, poor tolerance to physical activity and depression. In addition, alopecia, hair loss, delayed dentition, skin dryness and brittle nails may also occur after systemic or locoregional radiotherapy and as a result of chronic cutaneous GvHD (75).

In conclusion, in HSCT-survivors hormone replacement therapy with L-Thyroxine should be prescribed as a result of a thorough integrated assessment of thyroid function, complained symptoms and potential endocrine and non-endocrine coexisting clinical conditions.

#### 2.3.2 Autoimmune hyperthyroidism

Immune reconstitution after immunotherapy or discontinuation of immunosuppressants following HSCT may trigger abnormal dysimmune phenomena, including autoimmune hyperthyroidism (76). Accordingly, it is not infrequent that transplanted patients are started on synthetic antithyroid medication (ATM) before a completely retrieved immune function has been achieved.

Idiosyncratic neutropenia (absolute blood neutrophil count ≤ 1.5 × 10^9^/L) and agranulocytosis (neutrophils ≤ 0.5 × 10^9^/L in febrile or ≤ 0.1 × 10^9^/L in afebrile patients) represent rare but severe adverse effects of ATM, occurring mostly abruptly by the end of the third month after treatment start (77). Though no systematic analyses have assessed whether the risk or severity of these hematological side effects is greater after transplantation, it is reasonable to hypothesize that their onset early after HSCT may result in a longer-standing, deeper and potentially life-threatening stem cell aplasia compared to otherwise healthy patients. Given the idiosyncratic occurrence of agranulocytosis, clinicians should emphasize the importance of a prompt full blood count assessment in case of fever or signs of systemic infections.

Finally, ATM have been associated to an increased risk of abnormal liver function tests and, rarely, acute hepatitis (78). On the other hand, transplanted patients may present with prolonged hepatic toxicity *per se*, as a result of several competing factors, i.e. chronic liver GvHD, iron overload (as a result of multiple red blood cell transfusions), microangiopathy and iatrogenic hepatotoxicity of immunosuppressants and anti-infectious agents. Accordingly, a strict biochemical follow-up should be recommended in case of early ATM prescription, in order to promptly detect severe worsening of liver function markers.

## 3 Thyroid nodules and cancer

### 3.1 The epidemiology of thyroid nodules and cancer following HSCT

Pediatric HSCT survivors present a higher risk of developing secondary thyroid nodules and cancer than age-matched healthy individuals from the general population, with secondary thyroid carcinoma (STC) being the most frequent secondary neoplasia experienced following HSCT (79, 80).

Several studies have assessed the occurrence of thyroid nodules, either benign or malignant, among long-term survivors of pediatric HSCT, with a reported overall incidence ranging from 0 to 27%, due to differences in the composition of study cohorts, underlying diseases, conditioning regimens and duration of follow-up (18, 19, 26, 39, 57, 81). Interestingly, it has been reported that the cumulative incidence of nodules tends to be low in the first few years after HSCT, with a progressive increase as time elapses following transplantation, with a median time between HSCT and the detection of nodules ranging from 5 to 9.9 years (18, 19, 26, 57, 78, 79, 81, 82). Consistently, the detection of thyroid nodules has been reported as late as three decades following transplantation.

When specifically assessing the occurrence of STC, the reported incidence ranges from 0 to 14%, with papillary thyroid carcinoma as the most common hystotype, followed by follicular variants of papillary carcinoma. Notably, thyroid cancer tends to occur at a younger age among survivors of pediatric HSCT than in the general population (20 *versus* 45-50 years, in average) (26). In addition, post-HSCT STCs tend to show a more aggressive clinical course and a more frequently unfavorable presentation, characterized by larger tumor size and frequent extrathyroidal extension with lymphatic, perineural invasion and metastases (82).

HSCT-related risk factors for thyroid cancer include radiotherapy, exposure to alkylating agents, young age at transplantation and chronic GvHD.

### 3.2 Risk factors for thyroid nodules and cancer following HSCT

#### 3.2.1 Total body irradiation

The utmost sensitivity of the thyroid gland to the short and long-term effects of ionizing radiation exposure has been widely reported, especially in childhood and adolescence (36, 83). The pathogenesis of cancer following radiation exposure is well-established; radiotherapy may involve malignant transformation of irradiated cells as ionizing radiation can induce gene mutation by determining single and double strand DNA breaks (84), with the oncogenic potential of radiation being inversely related to DNA repair mechanisms efficiency (85).

Several analyses, effectively integrated in the setting of a huge-sampled pooled assessment by the British Childhood Cancer Survivor Study*,* have unveiled the mechanistic correlation between neck exposure to radiation during childhood and the occurrence of benign and malignant thyroid nodules (86–88). The mathematical relationship between the occurrence rates of secondary thyroid tumors and radiation doses involving the neck can be graphically represented by a parabolic model, with the hazard of STC directly increasing along with dosimetry for exposures as high as 29 Gy and with a drop of the risk above the threshold of 30 Gy. This mitigation phenomenon occurs as, for greater radiation doses, cell death by necrosis or apoptosis become preponderant, thus overcoming the risk of induced gene mutation and subsequent oncogenesis (89).

Moreover, it has been extensively demonstrated that a single acute exposure to radiation threatens the patient for a higher risk of cancer than a cumulative greater dose of radiation administered in smaller fractions (90, 91). As widely explained in *paragraph 2.2.1*, TBI has a pivotal role in the conditioning regimen preceding HSCT in children with haemato-oncological diseases, mainly represented by acute lymphoblastic leukemia. In order to minimize the deleterious effects of radiation exposure, current conditioning protocols including TBI suggest a total dose of 12 Gy administered in 6 to 8 fractions (92).

Despite these efforts and advances in the field of radioprotection, the 15-year cumulative incidence of STC following TBI reported in literature is as high as 27% (18, 81).

Many authors reported that the impact of TBI on nodule occurrence depends on age upon exposure, with the youngest patients being at highest risk for secondary neoplasia (77, 78, 93). Accordingly, Rizzo et al. demonstrated that the likelihood of any secondary neoplasia in children receiving radiation before 10 years of age was 55-folds higher than in the general population (93). In terms of STC, Cohen and colleagues showed that patients with a history of radiotherapy before 10 years of age had a three-fold increased risk of thyroid cancers (RR 3.44; CI 95%) (78).

The pathogenesis of papillary carcinomas following radiation has been historically assumed to result from activating rearrangements of the RET/PTC oncogene, overall detected at a much higher frequency in patients diagnosed with papillary thyroid carcinoma who have been exposed to ionizing radiation compared to unexposed patients (94, 95). More recently, radiation-induced gene fusions involving ALK and NTRK oncogenes have been reported as well (96–98). From a biological perspective, gene fusion-positive thyroid carcinomas are typically infiltrative tumors, with a higher degree of dedifferentiation, hence leading more frequently to first-line therapy resistance (88, 99).

#### 3.2.2 Chemo-only conditioning regimens

Though more frequently reported following TBI, thyroid nodules are also detected among HSCT survivors conditioned with chemotherapy alone (18, 38, 77, 78). Among antineoplastic drugs, alkylating agents are frequently administered both in the setting of front-line treatment of haematological malignancies and as a part of the conditioning regimens preceding HSCT. As their action is mediated by DNA damage involving both neoplastic and healthy cells, they may show a carcinogenic potential besides the desired cytotoxic effect.

The causative role of high-dose alkylating agents on the onset of either benign or malignant focal lesions involving the thyroid is still controversial. Indeed, the assessment of the contribution of single chemotherapeutic agents may be challenging and potentially misleading, as several antineoplastic medications are administered in the setting of polychemotherapy regimens undertaken frontline or as conditioning regimens before HSCT. Secondly, as radio- and chemotherapy are often combined within the therapeutic course of transplanted patients, the overwhelming radio-toxicity on thyroid gland may overshadow the carcinogenic effect of chemotherapy.

In terms of malignant thyroid tumors, data drawn from the CCSS support the hypothesis that alkylating agents and radiotherapy may play a synergic detrimental effect on the risk of developing STC among childhood cancer survivors (CCS) exposed to radiation doses as high as 20 Gy *plus* chemotherapy (100), while a borderline increase of the risk for thyroid neoplasia has been also recorded among patients receiving anthracyclines (89). Similarly, secondary thyroid carcinomas following chemotherapy have been reported by Oudin and colleagues in 2 not-irradiated patients *versus* 19 exposed to radiotherapy involving the neck directly or by scatter irradiation. In addition, Cohen et al. observed that 7 out of 32 patients developing STC had undergone radiation free conditioning regimens. Conversely, other studies assessing the results of long-standing endocrine follow-up among large cohorts of HSCT survivors report no cases of SCT in patients conditioned with TBI-free regimens (18, 26).

#### 3.2.3 Graft versus host disease and prolonged administration of immunosuppressants

Conflicting outcomes have been reported in the attempt to outline a potential mechanistical correlation between a previous medical history of acute or chronic GvHD and the onset of STC among HSCT-recipients.

In particular, Nelson et al. found no increased occurrence of second thyroid malignancies among patients with a previous history of acute GvHD, in a cohort of 674 patients, 17 of whom developed STCs (77). Similarly, Zubarovskaya and colleagues (19) did not outline a relationship between thyroid malignancy and chronic GVHD in a cohort of patients transplanted for ALL and exposed to TBI prior to HSCT. These results are consistent with those drawn by Bresters et al. (101), Vivanco et al. and Maeda et al. (81) in different study populations.

Conversely, Cohen et al. (77) observed a higher standardized incidence *ratio* (SIR) for secondary thyroid neoplasia among patients who had developed chronic GvHD, compared with those who had not (SIR, 9.68; 95%CI, 5.14 to 16.51), suggesting a possible association. Previously, Deeg et al. reported superimposable results (102).

Assessing the potentially independent detrimental effect of GvHD and immunosuppressants on the occurrence of thyroid disorders is extremely challenging. The demonstrated greater incidence of thyroid dysfunctions reported among patients treated with liver or kidney transplantation may support the negative role played by a long-standing history of immunosuppression on thyroid health, as these patients, treated with immunosuppressants, do not experience GvHD by definition (103). Conversely, the independent role of GvHD still needs to be clarified.

Finally, though the prolonged exposure to immunosuppressants to treat or prevent GvHD has been historically deemed as a potential causative factor in the development of thyroid cancer, this hypothesis has been widely challenged more recently (104).

#### 3.2.4 Demographical factors

Age at transplantation plays a key role on the likelihood of developing secondary thyroid tumor. Cohen and colleagues, who assessed the occurrence of STC among more than 70.000 transplanted patients, demonstrated that older age was protective over the occurrence of thyroid neoplasms. In particular, the authors found a 61-fold raised odd ratio among patients younger than 10 years at HSCT and a 9-fold increased odd ratio in the cohort of patients aged 11 to 20 years, irrespectively of the type of conditioning regimen received (78). Consistently, several other authors achieved superimposable results (77, 82).

The increased risk for STC in the youngest patients may be due to the great susceptibility to damage of the developing thyroid gland compared to the adult one.

On the other hand, there is no agreement on the predisposing role of gender on the development of STC following HSCT. Though several analyses identify male gender (77, 78) or female gender (38, 39) as potential detrimental factors, most studies did not observe any sex-related differences in the distribution of secondary thyroid cancer following transplantation (19, 81, 82).

#### 3.2.5 Coexistent non-HSCT-dependent risk factors among transplanted patients

As for functional thyroid disorders, several HSCT-unrelated risk factors experienced by transplanted patients need to be considered when assessing the overall risk of developing a STC following stem cell transplantation.

Firstly, in case of cancer-predisposing syndromes, the baseline clinical condition prompting the indication to perform HSCT may represent an independent risk factor, hence lowering the threshold for a dedicated follow-up irrespectively of the conditioning regimen undertaken before HSCT. For example, Fanconi Anemia may expose patients to an increased oncogenic risk *per se*, due to the failure in DNA interstrand crosslink repair mechanisms. Accordingly, a raised incidence of STC among transplanted patients with Fanconi Anemia has been reported (105).

A greater risk for secondary thyroid cancer among CCS has been variably described in patients diagnosed with Hodgkin lymphoma (HL) (100), severe aplastic anemia (SAA) or non-Hodgkin lymphoma (NHL), but none of these associations was confirmed upon multivariable analysis (77, 78). Other studies in the field did not outline any statistically significant relationships between the underline disease and the development of nodules, suggesting that, with the exception of cancer-predisposing syndromes, the risk for STC should be deemed as treatment-driven rather than related to the disease itself (18, 39).

Secondly, patients undergoing HSCT may have been previously exposed to prophylactic cranial or craniospinal irradiation as a part of the frontline treatment preceding transplantation. As the thyroid gland is located close to the treatment field, scattered radiotherapy targeting the cranium tangentially exposes thyroid to ionizing radiation (106). Similarly, thyroid may receive direct or scattered irradiation in the setting of locoregional irradiation delivered to patients diagnosed with haemato-oncological diseases, i.e. Hodgkin lymphoma (HL), or solid tumors involving the mediastinum, the chest or neck.

Finally, patients diagnosed with high-risk NBL may receive an autologous stem cell transplantation as a rescue after high dose chemotherapy. NBL patients undergoing a treatment with ^131^I-MIBG, have been demonstrated to be exposed to a higher risk of thyroid benign and malignant nodules (107–109).

### 3.3 Diagnosis and treatment of thyroid nodules: Peculiarities, lights and shadows among pediatric HSCT survivors

To the best of our knowledge, no guidelines specifically focused on the diagnosis and treatment of thyroid nodular findings following pediatric HSCT have been published to date. As for thyroid function disorders, though diagnostic and therapeutic solutions mirror the approach undertaken for the otherwise healthy general population (60, 110), we aimed at highlighting the specificities of survivors transplanted in childhood or adolescence, in order to guide clinicians in assessing and managing HSCT long-term *sequelae*.

Firstly, papillary thyroid carcinoma is the most common hystotype, both in the general population and among HSCT survivors. Nonetheless, it has been reported a higher incidence of pediatric follicular carcinoma among transplanted patients compared to otherwise healthy controls (78, 82, 111).

Lee et al. compared the clinicopathological features of 18 patients with post-transplantation secondary thyroid carcinoma with those of 723 patients diagnosed with primary differentiated thyroid carcinoma. Thyroid cancer in the transplanted cohort tended to be more aggressive than in the primary one, presenting with larger tumor size, more frequent extrathyroidal extension with lymphatic and perineural invasion and metastases (82).

Moreover, the irradiated group deserves a more invasive treatment approach. Indeed, *as per* American Thyroid Association guidelines, radical thyroidectomy rather than lobectomy should always be pursued following radiation exposure (110).

Radioactive iodine is used as adjuvant therapy in patients with thyroid cancer presenting with metastases, gross extrathyroidal extension and tumor size >4 cm (110). Though no recommendations specifically focused on the management of radiation-dependent STC are available, radioactive iodine ablation is commonly prescribed in order to reduce the risk for thyroid tumor recurrence (82). Nevertheless, radioiodine treatment has been described as potentially detrimental in term of risk of developing a third malignancy following STC, in particular among transplanted patients with HSCT-related or non-related cancer-predisposing conditions or other risk factors (112, 113). Accordingly, as the early detection of malignant nodules may result in a remarkable radioactive iodine-sparing approach, a strict follow up should be recommended to ensure a prompt detection and therefore a radical surgical treatment (108).

## 4 Thyroid health following HSCT: Practical recommendation for screening and management

As previously reported, thyroid *sequelae* may occur early following HSCT, but in most patients they may not become overt until decades into survivorship, mostly based on patient-specific causative factors and on the protocols undertaken (6, 73, 114, 115).

Accordingly, we believe that a tailored screening program for thyroid late effects should be pursued among childhood and adolescence HSCT survivors.

Though several *consortia* have provided recommendations for endocrine health surveillance among CCS (115–118), to the best of our knowledge no guidelines have thoroughly disclosed shared indications for screening and management of thyroid disorders among HSCT-survivors. Given the specificities of HSCT-related risk factors (conditioning regimens, prolonged and deep immunosuppression, GvHD and relative prophylaxis and treatment) and the growing body of non-malignant conditions for which stem cell transplantation has become the standard-of-care, we matched our aims to provide clinicians with accessible and handy recommendations, customized on the basis of the conditioning regimen administered and additional risk factors related or not to HSCT itself.

**Table 2** shows the recommendations for screening and management, by conditioning regimen and/or additional potentially detrimental factors.

**Table 2:** Recommendation for screening and management approach of thyroid disorders following haematopoietic stem cell transplantation, by conditioning regimen and/or additional potentially detrimental factors.

Conditioning regimen – treatment-related risk factors	Diseases most frequently involved	Monitoring for autoimmune or non-autoimmune thyroid function disorders	Monitoring for thyroid nodules/ secondary cancer	Debated subjects needing additional analyses
***Total body irradiation*** ***(10 to 18 Gy) without additional irradiation involving the neck***	***Acute lymphoblastic leukaemia; non-Hodgkin lymphoma***	***FreeT4, TSH*** - Assessed at least annually or earlier if clinically indicated1 - Starting from 1 year following HSCT - Both FreeT4 and TSH should be assessed, though the risk of central hypothyroidism is extremely low. - Patients with radiation-induced GH deficiency on treatment with recombinant human GH, TFT should be assessed more frequently (6-monthly). ***Anti-thyroid peroxidase, anti-thyroglobulin and/or anti-TSH receptor antibodies*** Not routinely required. Assessed in case of abnormal TFT or in case of sonographic findings consistent with of thyroiditis.	***Thyroid ultrasound versus neck palpation: thyroid ultrasound as a potential first choice*** As the risk of STC is remarkably higher following radiation, thyroid US allow early detection of malignant nodules and could be regarded as the first choice following TBI. Lifelong yearly assessment from 1 year after HSCT onwards. US screening is prescribed/ intensified in case of nodule detection. ***FNAB should be prescribed in case of suspicious sonographic findings***	***The benefits of thyroid ultrasound versus neck palpation still need to be clarified following HSCT.*** The following criteria should be considered: - clinician’s experience in detecting nodules by palpation (different specialists may be involved in long-term follow-up) - Age upon radiation: age <10 years expose patients to a remarkably higher risk of STC - Family history consistent with thyroid tumors. - Cancer-predisposing syndromes suspected or confirmed. - TBI delivered as single-fractioned dose exposes patients to a greater risk of secondary tumor ***The reliability of TI-RADS scoring*** *in prompting the prescription of FNAB for radiation-induced nodules needs to be clarified* ***The protective role of L-Thyroxin*** against the occurrence of second thyroid tumors in patients with subclinical hypothyroidism (TSH 5-to-10 microU/mL, normal FreeT4) is still debated.
***Total body irradiation*** ***(2 Gy) without additional irradiation involving the neck***	***Severe aplastic anemia***	***FreeT4, TSH*** - Assessed at least annually or earlier if clinically indicated1 - Starting from 1 year following HSCT. However, TFT should be assessed earlier among those patients exposed to ATG with signs or symptoms consistent with early-onset ATG-induced Graves’ disease or with clinically detected goiter. ***Anti-thyroid peroxidase, anti-thyroglobulin and/or anti-TSH receptor antibodies*** Not routinely required. Assessed in case of abnormal TFT or in case of sonographic findings consistent with of thyroiditis.	***Thyroid ultrasound versus neck palpation: thyroid ultrasound as a potential first choice*** As the risk of STC is remarkably higher following radiation, thyroid US allow early detection of malignant nodules and could be regarded as the first choice following TBI. Lifelong yearly assessment from 1 year after HSCT onwards. US screening is prescribed/intensified in case of nodule detection. ***FNAB should be prescribed in case of suspicious sonographic findings***	***The benefits of thyroid ultrasound versus neck palpation still need to be clarified following HSCT.*** The following criteria should be considered: - clinician’s experience in detecting nodules by palpation (different specialists may be involved in long-term follow-up) - Age upon radiation: age <10 years expose patients to a remarkably higher risk of STC - Family history consistent with thyroid tumors. - Cancer-predisposing syndromes suspected or confirmed. - TBI delivered as single-fractioned dose exposes patients to a greater risk of secondary tumor ***The reliability of TI-RADS scoring*** *in prompting the prescription of FNAB for radiation-induced nodules needs to be clarified* ***The protective role of L-Thyroxin*** against the occurrence of second thyroid tumors in patients with subclinical hypothyroidism (TSH 5-to-10 microU/mL, normal FreeT4) is still debated.
***Total body irradiation*** ***(10-18 Gy) with additional irradiation involving the neck (scatter irradiation following cranial/cranio-spinal irradiation or radiation delivered directly to the neck or the mediastinum)***	***Acute lymphoblastic leukaemia; non-Hodgkin lymphoma***	***FreeT4, TSH*** - Assessed at least annually or earlier if clinically indicated1 - Starting from 1 year following HSCT - Both FreeT4 and TSH should be assessed, especially among patients exposed to both cranial irradiation and TBI (hypothalamic-pituitary doses ≥ 25-30Gy). - Patients with radiation-induced GH deficiency on treatment with recombinant human GH, TFT should be assessed more frequently (6-monthly). ***Anti-thyroid peroxidase, anti-thyroglobulin and/or anti-TSH receptor antibodies*** Not routinely required. Assessed in case of abnormal TFT or in case of sonographic findings consistent with of thyroiditis. ***In case of radio-induced TSH deficiency:*** - the diagnostic role of brain MRI is debated. - The assessment of ACTH secretion is recommended before commencing treatment with L-thyroxin. - Consider a complete hypothalamic-pituitary function assessment	***Thyroid ultrasound versus neck palpation: thyroid ultrasound as a potential first choice*** As the risk of STC is remarkably higher following radiation, thyroid US allow early detection of malignant nodules and could be regarded as the first choice following TBI. Lifelong yearly assessment from 1 year after HSCT onwards. US screening is eventually intensified in case of nodule detection. ***FNAB should be prescribed in case of suspicious sonographic findings***	***The benefits of thyroid ultrasound versus neck palpation still need to be clarified following HSCT.*** The following criteria should be considered: - clinician’s experience in detecting nodules by palpation (different specialists may be involved in long-term follow-up) - Age upon radiation: age <10 years expose patients to a remarkably higher risk of STC - Family history consistent with thyroid tumors. - Cancer-predisposing syndromes suspected or confirmed - TBI delivered as single-fractioned dose exposes patients to a greater risk of secondary tumor - The overall dosimetry delivered to the thyroid. Given the high risk of STC following cranial/ locoregional radiation plus TBI, we deem thyroid US as highly recommended in this population ***The reliability of TI-RADS scoring in prompting FNAB in radiation-induced nodules needs to be clarified*** ***The protective role of L-Thyroxin*** against the occurrence of second thyroid tumors in patients with subclinical hypothyroidism (TSH 5-to-10 microU/mL, normal FreeT4) is still debated.
***Chemo-only conditioning including busulfan – no exposure to radiotherapy***	***Acute lymphoblastic leukaemia, acute myelogenous leukaemia, chronic myelogenous leukaemia, juvenile myelomonocytic leukaemia, non-Hodgkin lymphoma, primary immunodeficiency, myelodysplastic syndromes, inborn errors of metabolism***	***FreeT4, TSH*** - Assessed annually or earlier if clinically indicated1 - Starting from 1 year following HSCT, but can be postponed based on patients’ concurrent complications. - Alkylating agents are not associated with the risk of developing TSH deficiency. ***Anti-thyroid peroxidase, anti-thyroglobulin and/or anti-TSH receptor antibodies*** Not routinely required. Assessed in case of abnormal TFT or in case of sonographic findings consistent with of thyroiditis.	***Thyroid ultrasound versus neck palpation*** Thyroid palpation assessed annually from 1 year after HSCT onwards. Thyroid ultrasound can be performed based on local protocols as an alternative approach. US screening is prescribed or intensified in case of nodule detection. ***FNAB should be prescribed in case of suspicious sonographic findings***	***The benefits of thyroid ultrasound versus neck palpation still need to be clarified following HSCT.*** The following criteria should be considered: - clinician’s experience in detecting nodules by palpation (different specialists may be involved in long-term follow-up) - Family history consistent with thyroid tumors. - Cancer-predisposing syndromes suspected or confirmed ***The lower occurrence of thyroid function disorders following treosulfan compared to busulfan is debated*** Preliminary data support the hypothesis that Busulfan is the most thyroidotoxic agent, also when compared to treosulfan.
***Chemo-only conditioning not including busulfan – no exposure to radiotherapy***	***Acute lymphoblastic*** ***leukaemia, acute myelogenous leukaemia, chronic myelogenous leukaemia, juvenile myelomonocytic leukaemia, Hodgkin lymphoma, Non-Hodgkin lymphoma,*** ***hemoglobinopathy,*** ***primary immunodeficiency,*** ***myelodysplastic syndromes,*** ***myeloproliferative neoplasms,*** ***severe aplastic anemia and other bone marrow failure syndromes, hemophagocytic lymphohistiocytosis, inborn errors of metabolism***	***FreeT4, TSH*** - Assessed every 1-2 years or earlier if clinically indicated^1^ - Starting from 1 year following HSCT, but can be postponed based on patients’ concurrent complications. Low occurrence of hypothyroidism for the first 5 years following HSCT - Alkylating agents are not associated with the risk of developing TSH deficiency. ***Anti-thyroid peroxidase, anti-thyroglobulin and/or anti-TSH receptor antibodies*** Not routinely required. Assessed in case of abnormal TFT or in case of sonographic findings consistent with of thyroiditis.	***Neck palpation*** Thyroid palpation assessed annually from 1 year after HSCT onwards. US screening is prescribed in case of nodule detection. ***FNAB should be prescribed in case of suspicious sonographic findings***	***The lower occurrence of thyroid function disorders following treosulfan compared to busulfan is debated*** Preliminary data support the hypothesis that Busulfan is the most thyroidotoxic agent, also when compared to treosulfan.
***Patients exposed to ^131^I-MIBG, irrespectively of the conditioning regimen administered for autologous HSCT***	***Neuroblastoma***	***FreeT4, TSH*** - Assessed at least annually or earlier if clinically indicated^1^ - Starting from 1 year following exposure to ^131^I-MIBG ***Anti-thyroid peroxidase, anti-thyroglobulin and/or anti-TSH receptor antibodies*** Not routinely required. Assessed in case of abnormal TFT or in case of sonographic findings consistent with of thyroiditis.	***Thyroid ultrasound versus neck palpation: thyroid ultrasound as a potential first choice*** As the risk of STC is remarkably higher following ^131^I-MIBG, thyroid US allow early detection of malignant nodules and could be regarded as the first choice in this setting. Lifelong yearly assessment from 1 year after HSCT onwards. US screening is eventually prescribed or intensified in case of nodule detection. ***FNAB should be prescribed in case of suspicious sonographic findings***	***The benefits of thyroid ultrasound versus neck palpation still need to be clarified following HSCT.*** The following criteria should be considered: - clinician’s experience in detecting nodules by palpation (different specialists may be involved in long-term follow-up) - Family history consistent with thyroid tumors. - Cancer-predisposing syndromes suspected or confirmed

^1^ Signs and symptoms suggestive for thyroid disorders should be assessed upon every follow-up clinical examination. Hypothyroidism can be suggested by the onset of fatigue, daily somnolence, reduced tolerance to cold, constipation, unexplained weight gain, hoarseness, dry skin, depression, hypercholesterolemia, bradycardia, goiter. Signs or symptoms suggestive for hyperthyroidism are: anxiety, irritability, unintentional weight loss, tachycardia, palpitations, fine tremors of extremities, reduced tolerance to heat, irregular sleeping patterns, hair loss.

TBI, Total body irradiation; STC, secondary thyroid carcinoma; US, ultrasound; ATG - antithymocyte globulin; TFT, thyroid function tests; ^131^I-MIBG - iodine-131 meta-iodobenzylguanidine; FNAB, fine needle aspiration biopsy, TI RADS, Thyroid Imaging Reporting and Data System.

In terms of autoimmune or non-autoimmune thyroid function disorders, most guidelines published in the setting of childhood cancer survivorship recommend yearly clinical evaluation of symptoms and sign of hypo- or hyperthyroidism, together with serum free T4 and TSH assessment, for all those patients exposed to radiation involving the neck, starting from 1 year after the end of antineoplastic treatment (86, 119–121). As the analysis of the occurrence of thyroid function disorders following total body irradiation has shown a progressively increasing cumulative incidence as late as 30 years after HSCT (18, 26), a life-long yearly screening program is highly recommended among subjects exposed to TBI in childhood or adolescence. Consistently, the demonstration of superimposable rates in the incidence of thyroid function disorders among patients conditioned with busulfan- and TBI-based schedules should prompt clinicians to pursue a similar approach after chemo-only conditionings based on high-dose alkylating agents (18, 26). In detail, comparative studies assessing the detrimental role played by different chemo-only conditioning schedules shed light upon the need for a strict monitoring especially amongst patients conditioned with busulfan, that can be regarded as the most tyroidotoxic agent (18, 26).

Though an increased occurrence of autoimmune thyroid disorders has been reported after HSCT, a systematic monitoring of anti-thyroid antibodies is recommended neither among CCS, nor following HSCT (86, 119, 121). In detail, the widely described occurrence of adoptive autoimmunity as long as dysimmune phenomena recorded after stem cell engraftment may result in an overestimation of thyroid autoimmunity, as isolated positive autoantibodies inherited from the donor may be mere bystanders, not mirroring clinical and sonographic findings consistent with chronic thyroiditis or Graves’ disease. Accordingly, we recommend that anti-thyroid antibodies are checked once either abnormal thyroid function tests and/or or sonographic findings raised the clinical suspicion of chronic lymphocyte infiltration, hence prompting the indication of a thorough assessment including thyroid autoimmunity.

A combined evaluation of free T4 and TSH is warranted among patients exposed to TBI, for whom both the hypothalamic-pituitary-thyroid axis falls within the irradiation field (6, 119, 121, 122). Though infrequent after the low radiation dosimetry delivered by total body irradiation, central hypothyroidism has been described amongst irradiated transplanted patients, especially if previously exposed to cranial or cranio-spinal prophylaxis as a front-line treatment for acute lymphoblastic leukaemia or central nervous system tumors. Among childhood cancer survivors, the threshold for hypothalamic dosimetry above which the hazard of developing central hypothyroidism becomes overt is set at 30 Gy (89, 121). Nevertheless, several cases of TSH deficiency have been reported also following TBI alone, thus leading us to precautionally recommend a TSH and free T4 combined screening for all HSCT survivors.

Patients candidate to commence treatment with TKI or immunotherapy should be screened for thyroid function before being started on therapy and strictly (3-to-6 monthly) thereafter, as far as treatment is ongoing.

Similarly, children and adolescents treated with ATG or alemtuzumab, as a part of GvHD prophylaxis, should be screened for clinical and biochemical signs consistent with thyrotoxicosis early after HSCT, as the direct detrimental effect of both may result in early thyroid damage and subsequent development of thyrotoxicosis.

Among all the other transplanted patients, thyroid function disorders occur as late adverse effects, thus leading us to recommend for an annual assessment starting from 1 year following HSCT onwards (120, 121). We have previously demonstrated that the cumulative incidence of thyroid function disorders after chemo-only conditioning regimens not including busulfan tends to be extremely flat for the first 5 years following transplantation, thus allowing clinicians to eventually postpone the start of biochemical monitoring based on coexisting complications, unless clinically indicated (18).

The management of thyroid function disorders is overall superimposable to the approach undertaken in the otherwise healthy pediatric and young adult population. Nevertheless, as some authors have hypothesized that chronically elevated TSH values may play a potentially detrimental role for the development of secondary tumors following total body irradiation (123, 124), levothyroxine tends to be routinely prescribed among irradiated survivors with subclinical compensated hypothyroidism (normal freeT4, TSH between 5 and 10 microU/mL), aiming at TSH levels in the lower normal range. However, the clinical benefits of an interventional approach are still widely debated and challenged by other authors (119).

In terms of thyroid focal lesions, no shared protocols specifically focused on the diagnosis and management of thyroid benign or malignant nodules following pediatric HSCT are available to date. Current guidelines for the management of CCS by the Children’s Oncology Group recommend yearly neck palpation in order to screen for thyroid secondary neoplasia among patients exposed to radiotherapy involving the neck or following treatment with ^131^I-MIBG (121, 125). Nevertheless, a growing body of research has shed light on the greater screening value of thyroid ultrasound (US) in detecting and better characterize thyroid nodules among CCS, compared to clinical examination alone (6, 118).

An increasing number of retrospective studies and case reports advocate periodic thyroid US as the method of choice for screening cancer survivors and HSCT-recipients exposed to TBI or cranial-radiotherapy (6, 38, 81, 82, 118, 126). Some experts also recommend that the prescription of US as a screening tool in the setting of long-term survivorship is extended to all transplanted patients, regardless of the conditioning regimen undertaken, since there is some evidence that alkylating-based conditioning regimens may similarly increase the risk for secondary nodules (38).

Comparing neck palpation to thyroid US in a case report series, Gabby Atlas et al. have recently pointed out the remarkably higher sensitivity rates of the latter in detecting thyroid nodules, leading to a precocious identification of focal lesions at risk for malignancy (126). Similarly, Cohen and colleagues and Lee et al. observed that in about half of their transplanted cohort with STC (78, 82), neither neck palpation nor biochemical results would have suggested the finding of secondary thyroid cancer, thus leading to a remarkable diagnostic delay without a systematic US screening. Accordingly, neck palpation alone may result in a more invasive treatment and in greater procedure-related risks.

On the other hand, benign thyroid nodules are not infrequent also in the general population and are even more commonly found among irradiated patients, as radiation may alter thyroid parenchyma and structure (81, 110). Accordingly, the use of thyroid ultrasound as a screening method may determine an overdiagnosis of nodules, without providing unique keys to discriminate between benign and potentially malignant lesions. Indeed, although thyroid US presents a specificity and sensibility as high as 95-100% for nodule detection (127, 128), no single sonographic feature allows radiologists to discriminate between benign nodules and STC (118, 129, 130). Moreover, though several scoring systems assessing combined US features have been proposed to differentiate malignancy from indolent nodules, there is no agreement on their accuracy, particularly in the irradiated population (118, 131, 132). Finally, as stated by the American Thyroid Association, the evaluation and treatment of nodules sized <1 cm is potentially unfavorable under a cost/effectiveness perspective, as there is no evidence of a real advantage of the treatment of small tumors. Therefore, it should be considered that a systematic US screening may identify more nodules than neck palpation, but this may potentially result in unnecessary more invasive investigations and anxiety for patients and families.

Overall, as both neck palpation and ultrasound screening approach present with advantages and disadvantages, the identification of additional risk factors among recipients (age <10 years upon HSCT, baseline cancer-predisposing syndromes, exposure to radiotherapy involving the neck prior to transplantation and TBI-based conditioning regimens) should be regarded as pivotal elements in outlining an integrated customized risk assessment and identifying the most suitable screening approach (118) (**Table 2**). Additionally, as different specialists may be involved in the long-term follow up of transplanted patients, clinician’s expertise in detecting nodules by palpation should be considered in order to choose the best screening modality.

The screening program among transplanted patients is still a topic of debate, as different studies suggest annual *versus* bi-annual surveillance, depending on local protocols (26, 118). Also, another subject for discussion is the duration of follow-up. Different reports have documented that the longest the follow-up is, the highest is the probability to early detect a thyroid potentially malignant nodule. In particular, Oudin et al. showed an incidence of thyroid cancer as high as 1.7% at 10 years of follow-up, which rose to 9.6% at 20 years (38), while Vivanco et al. outlined a far higher cumulative incidence of nodules 15 years compared to 10 years after TBI, suggesting the need to extend the duration of follow-up (81). Overall, published data support the indication of a life-long monitoring of thyroid health among transplanted patients, particularly for those exposed to TBI or radiation involving the neck.

The rationale for prescribing periodic US is to detect thyroid nodules at a very initial stage and to identify those deserving additional investigations. In common clinical practice, in the general population without specific risk factors, nodules are classified according to sonographic criteria based on shape, composition, echogenicity, presence of calcifications, margins, and location in order to assess the overall risk of malignancy. Several tools and algorithms (such as the Thyroid Imaging Reporting and Data System, TIRADS score) have been developed, in order to provide clinicians with standardized scoring systems that may guide them in assessing the need for further investigations (fine needle aspiration biopsy, FNAB) (133).

Overall, four different *consortia* have developed risk stratification reporting systems to assess the likelihood of malignancy when a thyroid nodule is detected in adulthood: the American College of Radiology (ACR-TIRADS), the European Thyroid Association (EU-TIRADS), the Korean Society of Thyroid Radiology (K-TIRADS) and the American Thyroid Association (ATA). The strengths and weaknesses of each scoring system have already been widely outlined (134). The diagnostic accuracy of K-TIRADS has been recently assessed among 107 patients and it has resulted in the lowering of the threshold size for suggesting biopsy from ≥1.0 cm to ≥0.6 cm for K-TIRADS 4 and ≥0.5 cm for K-TIRADS 5 (135).

Some authors speculate that these criteria are not appropriate to characterize nodules detected in patients treated with either TBI or regional RT, as radiation may induce a shrinkage in thyroid volume and possibly in the size of nodules, turning the dimensional parameter into an unreliable criterium (18, 111). Therefore, a prompt indication to FNAB for suspicious nodules, even those smaller than 10 mm in diameter, should be regarded as a reasonable approach, especially among cancer survivors treated with TBI or regional RT (126), especially if <10 years upon exposure or if they present with enlarged cervical lymph nodes (18, 118).

The reporting systems for pediatric thyroid nodules should be improved including future large studies with validation cohorts of US criteria (136).

Any indeterminate (Bethesda III/IV/V) pediatric FNAB is generally referred for surgery because rates of malignancy were reported to be higher in pediatric cohorts than adults. However, possible ancillary tools may change the clinical behavior, allowing in the future a more conservative approach. A recent study explored the use of artificial intelligence to better determine which pediatric thyroid nodules warrant FNAB and surgical intervention, with the intent to avoid surgery for benign lesions (137).

It has long been debated whether thyroid dysfunction could be a red flag for the occurrence of thyroid nodules. While some authors have suggested a relationship between raised TSH levels and the development of thyroid cancer in the general population (138, 139) latest studies agree on the lack of a systematic association between functional thyroid disorders and secondary thyroid cancer among HSCT patients (38, 78). Therefore, the development of a malignant nodule is not predictable by thyroid function tests.

Finally, regarding therapeutic approaches, the treatment of STC after transplantation do not differ from the one received by CCS for primary thyroid neoplasia. Lee et al. observed that post-HSCT thyroid cancers are more aggressive than the primary ones; indeed, most patients underwent total thyroidectomy combined with radioactive iodine ablation (82).

While surgery may be mostly required, an early diagnosis may reduce the use of radioactive iodine, which would expose the patients to an additional risk to develop a third malignancy (112, 113).

## Data availability statement

The original contributions presented in the study are included in the article/supplementary material. Further inquiries can be directed to the corresponding author.

## Author contributions

AC, ABa and ABi conceived the idea of the present manuscript. BR, SDM, MA, MF, VG, FM, MN, CP, ML performed the systematic revision of the literature. AC, SM and MN supervised the systematic revision of the literature and drafted the first version of the manuscript. AC and SM drafted the tables. ABa, ABi and FP critically revised the manuscript. All the authors agree with the final version of the manuscript.
